# The Prognostic Significance of Uric Acid/Albumin Ratio in Patients with Aortic Stenosis Following Transcatheter Aortic Valve Implantation for Major Adverse Cardiac and Cerebral Events

**DOI:** 10.3390/medicina59040686

**Published:** 2023-03-30

**Authors:** Halil Ibrahim Biter, Aydin Rodi Tosu

**Affiliations:** Sultangazi Haseki Training and Research Hospital, Istanbul 34260, Turkey

**Keywords:** TAVI, uric acid, albumin, MACCEs

## Abstract

*Background*: The goal of this study was to examine if the uric acid/albumin ratio (UAR) could predict major adverse cardiac and cerebral events (MACCEs) such as stroke, readmission, and short-term all-cause death in aortic stenosis (AS) patients, after transcatheter aortic valve implantation (TAVI). *Material and Methods*: A total of 150 patients who had TAVI for AS between 2013 and 2022 were included in our study, retrospectively. Before the TAVI, each patient’s baseline uric acid/albumin was determined. The study’s major endpoint was MACCEs, which included stroke, re-hospitalization, and 12-month all-cause death. *Results*: The UAR was found to be higher in TAVI patients who developed MACCEs than in those who did not develop them. Multivariate Cox regression analysis revealed that the UAR (HR: 95% CI; 2.478 (1.779–3.453) *p* < 0.01), was an independent predictor of MACCEs in AS patients after TAVI. The optimal value of the UAR for MACCEs in AS patients following TAVI was >1.68 with 88% sensitivity and 66% specificity (AUC (the area under the curve): 0.899, *p* < 0.01). We noted that the AUC of UAR in predicting MACCEs was significantly higher than the AUC of albumin (AUC: 0.823) and uric acid (AUC: 0.805, respectively). *Conclusion*: MACCEs in AS patients who received TAVI may be predicted by high pre-procedural uric acid/albumin levels. The uric acid/albumin ratio (UAR) can be used to determine MACCEs in such patients following TAVI because it is inexpensive and straightforward to calculate inflammatory parameters.

## 1. Introduction

In older people, aortic valvular anomalies are relatively common. The condition was shown to have a small male predominance. Only 2% of all cases had severe aortic stenosis [1]. Aortic stenosis begins with a plaque similar to coronary artery disease [2]. Age, male sex, hyperlipidaemia, and signs of active inflammation are risk factors associated with both diseases that have been linked to coronary artery disease [3].

Transcatheter aortic valve implantation (TAVI) is presently an alternate therapy option to surgically implanted aortic valve implantation in inoperable and high-risk patients with severe symptomatic AS [4]. A greater baseline inflammatory status as evaluated by C-reactive protein (CRP) levels may predict increased intermediate-term mortality following TAVI, according to a meta-analysis of 14 trials involving 3449 TAVI patients [5].

An antioxidant called uric acid (UA) can eliminate more than half of the free radicals in human blood [6]. It also has a significantly stronger antioxidant capability than vitamins C and E [7]. According to several studies, exogenous administration of uric acid was reported to be neuroprotective in several models of transient focal brain ischaemia in rats [8]. Furthermore, earlier studies have demonstrated that serum albumin possesses strong antioxidant effects in the face of oxidative stress [9,10].

Serum albumin (SA), the most well-known and vital protein in human serum. Serves a variety of physiological activities. Reduced synthesis and higher catabolism of SA have been linked to an enhanced inflammatory response. Lower SA levels have been linked to increased blood viscosity and endothelial dysfunction [11].

As a result, UA and albumin both represent the body’s antioxidant status and lower total oxidative stress. The connection between antioxidant status and AS after TAVI is still unknown, though. In order to determine whether there was a connection between the UA/A ratio (UAR) and MACCEs in AS after TAVI, the aim of this study was to use a hospital-based investigation.

## 2. Material and Methods

### 2.1. Study Population

150 patients with severe AS symptoms who underwent TAVI at a tertiary cardiac hospital between 2013 and 2022 were included in this retrospective research. The remaining 150 patients with a life expectancy of at least a year who were deemed to be at high risk based on clinical evaluations by a multidisciplinary heart team made up the trial population after the exclusion criteria. The subsequent parameters were used as exclusion standards: (I) an acute or ongoing infection, (II) a systemic inflammatory or autoimmune condition, (III) a history of liver disease (liver function parameters greater than three times the upper normal limit), (IV) any clinically significant endocrine, haematological, respiratory, or metabolic condition, and (V) cancer. Clinical and laboratory data were gathered for all patients and entered into the hospital’s electronic database. Each patient provided informed consent prior to TAVI. The Society of Thoracic Surgeons Score (STS) risk model was used to select those with significant symptoms of AS based on anticipated perioperative or short-term mortality.

The risk of death following the index TAVI surgery was calculated by the heart team using recommendations based on a risk model created by the EuroSCORE II (ES II). Using online calculators (www.euroscore.org and https://tools.acc.org/tavrrisk, accessed on 25 December 2022), the EuroSCORE II scores were determined. The transfemoral access for TAVI was the standard route; the transapical, carotid, and subclavian routes were never used. The heart team made the valve selection at their discretion.

The procedure was performed in the cardiac catheterisation laboratory under general anaesthesia or sedoanalgesia with transoesophageal echocardiography guidance. All patients received aspirin (81 mg) and clopidogrel (≥300 mg) before the procedure and heparin during the procedure; patients continued to take aspirin indefinitely and clopidogrel for a minimum of 1 month. After the index procedure, all patients were followed up at 30 days, 6 and 12 months.

A patient with severe AS was deemed a candidate for TAVI after being identified as having a high or very high risk of undergoing heart surgery. Procedure concerns were found using the valve academic research consortium 2 (VARC-2) criteria. Patients with and without MACCEs were separated into two groups for the study [12]. The local ethics committee reviewed our working procedures and approved it on 21 December 2022 with decision number 223-2022.

### 2.2. Uric Acid/Albumin Ratio

All patients had blood drawn from the antecubital vein prior to TAVI. The UA/A ratio was calculated by dividing uric acid by albumin.

### 2.3. Definitions

According to the Valvular Academic Research Consortium (VARC) criteria [12], MACCEs were determined to include all short-term deaths at 12 months, re-hospitalization, pacemaker necessity, significant bleeding, major vascular complications, stroke or transient ischemic attack. To assess and confirm medium-term deaths, a state-wide death registration database as well as the hospital’s computerized database were used. A TIA or a stroke was defined as a neurological dysfunction lasting less than 24 h. Readmission within 30 days was considered as re-hospitalization.

### 2.4. Statistical Analysis

While categorical data were reported as percentages, continuous variables were presented as means and standard deviations if they were normally distributed, and medians (interquartile ranges (IQRs)) if they were not. The Kolmogorov–Smirnov test was used to determine whether categorical variables were normally distributed and the Chi-square test was performed to compare categorical variables between the groups. Depending on whether the continuous variables were regularly distributed or not, a Student’s *t*-test or Mann–Whitney U test was employed to compare the continuous variables between the groups. Variables associated at a *p* < 0.05 level in univariate analysis were included in the multivariate cox regression analysis to identify the independent predictors of the one-year MACCEs. The results were presented as hazard ratios (HR) and 95% confidence intervals (CI). The receiver operating characteristic (ROC) curve, area under the curve (AUC), and 95% confidence interval were used to examine the discriminatory power of UAR, uric acid, and albumin in identifying the one-year MACCEs. The maximum sensitivity and specificity point served as the starting point for the calculation of the ideal cut-off value. The value of *p* < 0.05 was chosen as the statistical significance criterion. The Statistical Package for the Social Sciences, version 24.0, was used to conduct all statistical analyses. (IBM Corp., Armonk, NY, USA).

## 3. Results 

The study population’s median age was 77 ± 5 years, and 84 patients (or 56%) were men. Patients with MACCEs had worse NYHA class (*p* = 0.016) and higher cardiovascular disease history (*p* = 0.003). Patients with MACCEs exhibited lower valve area and a larger mean gradient when it came to the echocardiographic measures (*p* = 0.022 and *p* = 0.002, respectively). Table 1 summarizes the demographic, clinical, and laboratory details of the research population.

There were no differences in the groups’ aortic predilatation, postdilation, implantation depth, or type of valve (balloon-expandable or self-expandable). Major vascular complication rate was 17.1%, bleeding complication rate was 22%, permanent pacemaker requirement was 17.1%, postprocedural TIA/IS rate was 12.2%, rehospitalization rate was 29.3%, and all-cause mortality rate was 24.4% in the group developing MACCE. The procedure and post-procedural parameters for the study population during the follow-up period are reported in Table 2.

We conducted both univariable and multivariable Cox regression analysis to identify the determinants of MACCEs. The vascular disease history (VDH), aortic valve area (AVA), Major vascular complications (MVC), serum albumin, uric acid and UAR predicted the MACCEs in univariable regression analysis. We didn’t analyse uric acid and albumin along with the UAR parameter in the multivariate regression analysis as uric acid and albumin are components of UAR. For this reason, we performed two separate regression analyses.

In the multivariable Model-1 Cox regression analysis, CRP (HR = 1.087, *p* = 0.015) uric acid (HR = 1.337, *p* < 0.001) and albumin (HR = 0.396, *p* < 0.001) were independent predictors for MACCEs. Moreover, multivariable Model-2 Cox regression analysis revealed that CRP (HR = 1.096, *p* = 0.007) and UAR (HR: 2.478, *p* < 0.001) independently predicted the development of MACCEs (Table 3).

**Figure 1:**
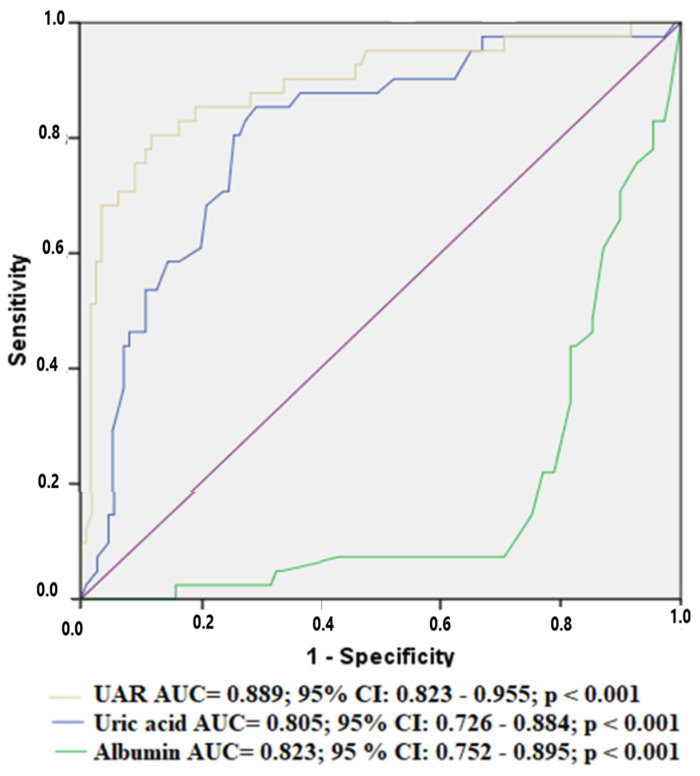
Discriminatory performances of UAR, uric acid and albumin for MACCEs.ROC Curve.

In a ROC curve analysis, UAR exhibited better discriminative performance (AUC: 0.889, *p* < 0.001) than uric acid (AUC: 0.805, *p* < 0.001) and albumin (AUC: 0.823; *p* < 0.001) (Figure 1). In addition, a cut-off value of the UAR in predicting the MACCEs was determined to be greater than 1.68, with a sensitivity of 88% and a specificity of 66%.

## 4. Discussion

This is the first study to examine whether UA/albumin levels in AS patients receiving TAVI can predict unfavourable cardiac and cerebrovascular outcomes. Elevated UA/albumin levels have been associated with a higher risk of MACCEs.

AS is now recognized as the outcome of intricate biological processes controlled by valve interstitial cells, fibroblast-like cells that predominately fill the aortic valve. Endothelial injury, lipid accumulation, chronic inflammatory infiltrates, and microcalcification are the hallmarks of valve lesions [13].

The prognosis for severe aortic stenosis is depressing, with congestive heart attack and angina stroke occurring. According to the conventional view, surgical AVR provides the highest chance to deliver and dispose of long-term care. However, approximately one-third of patients with severe aortic stenosis measures are not suitable for surgery because of comorbidities and surgical risk. In the experience of early transcatheter aortic valve implantation (TAVI) for over/high purposes for surgical aortic valve replacement (SAVR), the global mortality rate at one year was as high as 25%. Since then, patients with moderate and low rates have had access to TAVI, and the number of intensive surgeries annually has increased significantly. 

The valvular endothelium is assumed to have been damaged by mechanical stress or other risk factors in the initial incident, allowing for inflammatory cell recruitment and lipoprotein infiltration and retention. Because of a shift in therapeutic paradigm toward minimally invasive therapies, TAVI has transformed clinical outcomes in AS, particularly in those who were previously considered inoperable.

Selective candidate criteria, as well as advancements in TAVI operational procedures, are key factors in achieving positive results. However, the one-year mortality rate after TAVI is still significant and exceeds 15% in current practice. Acute renal failure, severe postoperative paravalvular leak, and comorbidities such as chronic obstructive pulmonary disease (COPD), heart failure, chronic kidney disease (CKD) and previous strokes have all been associated with higher mortality rates over time [14].

Geriatric adults with aortic valve stenosis (AS) are especially vulnerable to cardiovascular disease. Low mobility, muscle weakness, cachexia, and other frailty signs characterize a considerable number of these patients. Additionally, it is believed that the development of a systemic inflammatory response syndrome (SIRS) after TAVI is connected to early mortality. Two pro-inflammatory markers, CRP and IL-6 have been connected to aging, multimorbidity, and late-life disability. Following inflammatory stimulation, monocytes and macrophages are thought to be the primary producers of cytokines [15,16]. 

In some clinical disorders, adverse clinical events are more common in the chronic inflammatory state. According to literature evidence, increased inflammation, as in coronary atherosclerosis, is detrimental to cardiac endothelium and valve tissue. As a result, heart valve degeneration can be observed more clearly, and the prognosis may worsen by progressing more rapidly. Even when the valvular condition is treated, a higher inflammatory condition is associated with a higher risk of death. Risk ratings and serum biomarkers for congestive heart failure and other conditions were reviewed in patients with TAVI to see how well they performed. However, there is no commonly used unique method for patient death after TAVI. Increased levels of inflammatory biomarkers before and after TAVI can help predict how well patients respond to the procedure [14,15,16].

Hyperuricemia and cardiovascular disease are strongly linked, according to epidemiological studies [17]; higher UA levels are an independent and statistically significant predictor of cardiovascular disease and mortality [18]. UA may act as a selective antioxidant and free radical scavenger in the physiological settings, protecting endothelium from oxidative stress injury [19]. On the other hand, numerous animal studies have demonstrated that elevated serum UA levels are detrimental to the cardiovascular system and lipid metabolism. Endothelial cells’ activation of the renin–angiotensin system (RAS) and reduction in insulin-induced nitric oxide (NO) synthesis were the main contributors to this adverse effect [20].

The most prevalent plasma protein, albumin, is an adverse acute-phase reactant. Its blood content decreases when there is inflammation, and it is produced by the liver. Albumin had previously been linked to the severity of the inflammation, the prognosis of the illness, and death [21,22,23]. According to Wada et al., low albumin and high CRP levels were associated with an increase in cardiovascular events [24]. Each inflammatory state may cause various responses from acute-phase reactants. Combining albumin and CRP into a single index as an inflammation-based prediction score provides stability between CRP and albumin levels in illnesses where inflammation is considerable. According to a study that examined CARs’ (CRP/Albumin ratio) impact in critically ill patients, the elderly, particularly those with acute exacerbations of chronic diseases, have a high predictive value for the treatment [25]. According to Okuno et al., patients with lower PNI (prognostic nutritional index) and higher Controlling Nutritional Status scores had a statistically significant higher 1-year mortality rate as well as composite outcomes of mortality and re-hospitalization due to heart failure [26]. Serum levels are used by the PNI to assess nutritional and inflammatory status. We are also aware that vascular injury, renal injury, and the concentrations of different cytokines all affect serum albumin levels. Moreover, it plays a significant part in regulating serum electrolyte levels and has antioxidant properties. Free fatty acids and steroid hormones can also interact with it. It is highly challenging to pinpoint the precise mechanism of the association between serum albumin level and mortality due to these numerous functions, in addition to the fact that it serves as a marker for mortality.

In a number of clinical disorders, adverse clinical events are more frequently observed in the chronic inflammatory state. Increased inflammation, as found in coronary atherosclerosis, is detrimental to the cardiac endothelium and valve tissue, according to literature evidence. As a result, cardiac valvular degeneration can be observed more clearly and may progress more rapidly with worse prognoses. Even when the valvular condition is treated, a higher inflammatory state is linked to a higher risk of mortality [14,27,28].

The presence of a proinflammatory condition before the TAVI procedure has been shown in many studies to be associated with an increase in long-term mortality and complication rates after the procedure. In the study of Hirofumi et al., CRP levels were studied before the TAVI procedure and high serum CRP level on admission was significantly associated with an increased risk of all- cause death after TAVI, particularly within the first 3 months after TAVI was found.

Our study shows that serum uric acid and albumin are clinical biomarkers that are easily accessible and timely with little additional cost to healthcare, and their combination may become a suitable index to evaluate the risk of MACCE’s in patients who have had TAVI. This discovery is especially helpful in some clinical situations, such as impoverished locations or settings requiring frequent follow-up, where access to medical technological support is limited.

In recent studies, UA/A as an inflammatory marker has been used to predict mortality risk in acute kidney injury and acute coronary syndromes. Ozgur et al., in their study, determined that the UA/A ratio was associated with an increased risk of 30-day mortality in patients who developed AKI. That the albumin levels of patients whose mortality was recorded at 30-day follow-up were significantly lower than those who survived their hospitalization and that the most likely cause was malnutrition, but they stated that there is no evidence to support albumin replacement to treat hypoalbuminemia in order to increase survival rates [27]. Cakmak et al. suggested UA/A is a superior marker than CAR for predicting the extent and severity of CAD in patients with NSTEMI. They showed that although CAR independently predicted CAD severity, UAR exhibited better independent prediction performance compared to CAR. In patients undergoing PCI, hypoalbuminemia with high uric acid has been found to cause a synergistic side effect on the long-term risk of major cardiovascular events [29]. Again Shunbao Li et al.; showed in their study that the UA/A value at admission is an independent predictor of long-term cardiac mortality after percutaneous coronary intervention in patients with unstable angina pectoris [30].

We think that the underlying mechanism is a shift in the inflammatory condition, which correlates with patient fragility before TAVI and seems to increase mortality after valve implantation. The current study may be the first to show that UAR in AS patients who had TAVI predicts MACEs. The UAR is a straightforward inflammatory index that can be computed using whole blood count information. According to our research, AS patients are more likely to experience negative cardiac and cerebrovascular outcomes when their UAR is raised after TAVI. These findings imply that having an increased inflammatory status as measured by UAR is a good predictor of MACCEs after TAVI. Furthermore, our data imply that patients with a high UAR after TAVI should be closely monitored. In addition, in our study, similar to the previous meta-analysis [5], it was observed that a higher baseline inflammatory state measured by C-reactive protein (CRP) levels may herald an increase in mid-term mortality following TAVI and predict an increase in MACCE’s.

Finally, our findings supported UAR’s predictive value for increased mortality after TAVI. Age, nutritional status, diabetes, and CVD history may all have an impact on this negative effect. More investigation is required to determine the underlying mechanism.

## 5. Conclusions

Our findings suggest an independent role of UA/A in early mortality in patients with isolated severe degenerative AS after TAVI beyond conventional inflammatory markers. From a clinical standpoint, we think that preoperative UA/A is an easy, inexpensive and promising predictive inflammatory parameter and may be a part of the cardiovascular examination to identify high-risk individuals for the TAVI procedure. UA/A could assist clinicians in their decision-making and may advise individual patients of their risk due to its ability to predict early mortality in patients undergoing TAVI. However, to evaluate the predictive value of UA/A, and especially its prognostic value in patients who underwent TAVR, large-scale and prospective studies are still required.

## 6. Study Limitations

Our research has some limitations. To begin, this study is both retrospective and observational in nature. Second, the sample size in this study is small and relied on the experience at a single centre. Because of this, the study’s generalizability may be constrained by the small number of patients who received TAVI. Third, in the current study, long-term mortality was estimated using a spot laboratory value.

## Figures and Tables

**Table 1:** Baseline demographic, clinical and laboratory parameters of the study cohort grouped according to the presence of MACCEs.

Variables	All Population	No MACCEs (n = 109)	MACCEs (n = 41)	*p*
Male gender, n %	84 (56)	57 (52.2)	27 (65.8)	0.136
Age	77 ± 5	77 ± 5	77.1 ± 4	0.903
BMI (kg/m^2^)	26.8 ± 4.2	26.7 ± 4.0	27.0 ± 4.8	0.673
Hypertension, n (%)	90 (60)	66 (60.5)	24 (58.5)	0.820
Diabetes mellitus, n (%)	47 (31.3)	30 (27.5)	17 (41.5)	0.101
Vascular disease history, n (%)	41 (27.3)	20 (19.8)	21 (42.9)	0.003
Chronic renal failure, n (%)	25 (16.7)	17 (15.6)	8 (19.5)	0.566
COPD, n (%)	56 (37.3)	39 (35.8)	17 (41.5)	0.521
Smoking, n (%)	20 (13.3)	15 (13.8)	5 (12.2)	0.801
CVA history, n (%)	11 (7.3)	6 (5.5)	5 (12.2)	0.161
NYHA Class III-IV, n (%)	10 (6.7)	4 (3.7)	6 (14.6)	0.016
Atrial fibrillation, n (%)	18 (12.0)	13 (11.9)	5 (12.2)	0.964
Presence of RBBB or LBBB, n (%)	21 (14)	14 (12.8)	7 (17.1)	0.506
Aortic valve area, cm^2^	0.81 ± 0.10	0.82 ± 0.1	0.78 ± 0.1	0.022
Mean Aortic valve gradient, mmHg	47.9 ± 4.2	47.3 ± 3.6	49.6 ± 5.0	0.002
Left ventricular ejection fraction, %	54.8 ± 8.5	55.3 ± 7.6	53.4 ± 10.6	0.223
Fasting glucose, mg/dL	123.8 ± 39.4	124.2 ± 40	122.7 ± 38.4	0.832
Creatinine, median, mg/dL, [IQR]	0.90 [0.80–1.50]	0.90 [0.70–1.50]	0.90 [0.80–1.50]	0.948
TC, mg/dL	209 ± 39	208.7 ± 43.7	208.6 ± 34	0.984
Triglyceride, mg/dL, median, [IQR]	175 [150–195]	175 [148–195]	175 [153–195]	0.432
WBC, cells/mcL	6.9 ± 1.7	6.4 ± 1.7	7.9 ± 1.1	<0.001
Hgb, g/dL	15.2 ± 3.8	15.3 ± 4.3	14.9 ± 1.0	0.504
Platelet, cells/mcL	263 ± 71	260 ± 68	269 ± 79	0.499
CRP, median, mg/L, [IQR]	3.50 [1.28–6.30]	3.2 [1.0–4.9]	5.7 [2.9–10.0]	0.001
Uric acid, mg/dL	6.0 ± 2.1	5.4 ± 1.9	7.5 ± 1.9	<0.001
Albumin, g/dL	3.4 ± 0.79	3.7 ± 0.6	2.7 ± 0.8	<0.001
UAR	1.92 ± 0.96	1.51 ± 0.58	2.99 ± 0.92	<0.001

Continuous variables were presented as means ± standard deviations if normally distributed and medians (interquartile ranges (IQRs)) if not normally distributed, while categorical variables were given as percentages. Abbreviations: BMI, body mass index; TC, total cholesterol, CRP, C-reactive protein; UAR, uric acid to albumin ratio.

**Table 2:** Procedural and postprocedural parameters of study population during the follow-up period.

Variables	All Population	No MACCEs (n = 109)	MACCEs (n = 41)	*p*
Conscious sedation, n %	85 (56.7)	65 (59.6)	20 (48.8)	0.232
Type of Valve, n (%)				0.309
SEV	74 (49.3)	51 (46.8)	23 (56.1)	
BEV	76 (50.7)	58 (53.2)	18 (43.9)	
Predilatation, n (%)	46 (30.7)	34 (31.2)	12 (29.3)	0.820
Postdilatation, n (%)	21 (14)	16 (14.7)	5 (12.2)	0.696
Implantation depth, mm	5.2 ± 0.8	5.2 ± 0.7	5.3 ± 1.0	0.445
Paravalvular leakage (>2+), n (%)	11 (7.3)	6 (5.5)	5 (12.2)	0.161
Major vascular complications, n (%)	7 (4.7)	0 (0)	7 (17.1)	<0.001
Bleeding complications, n (%)	9 (6)	0 (0)	9 (22)	<0.001
Pericardial Tamponade, n (%)	5 (3.3)	3 (2.8)	2 (4.9)	0.518
Acute renal failure, n (%)	13 (8.7)	9 (8.3)	4 (9.8)	0.771
Permanent pacemaker, n (%)	7 (4.7)	0 (0)	7 (17.1)	<0.001
Postprocedural IS or TIA, n (%)	5 (3.3)	0 (0)	5 (12.2)	<0.001
Rehospitalization, n (%) (cardiovascular-caused)	12 (8)	0 (0)	12 (29.3)	<0.001
Sepsis with worsening of heart function, n (%)	0	0	0	0
Poor positioning of the prosthesis/thrombosis, n (%)	6 (4)	3 (2.8)	3 (7.3)	0.204
Myocardial infarction, n (%)	0	0	0	0
Infective endocarditis, n (%)	0	0	0	0
Death (all-cause), n (%)	10 (6.7)	0 (0)	10 (24.4)	<0.001

Categorical variables were given as percentages. Abbreviations: SEV, self-expandable valve; BEV, balloon expandable valve. IS, ischemic stroke; TIA, transient ischemic attack.

**Table 3:** Factors that were found to be independently associated with the MACCEs in univariate and multivariate regression analysis models.

Variables	Univariate HR (95% CI)	*p*	Model 1 Multivariate * HR (95% CI)	*p*	Model 2 Multivariate * HR (95% CI)	*p*
VDH	0.422 (0.187–0.953)	0.038	1.793 (0.923–3.485)	0.085	1.838 (0.984–3.545)	0.056
AVA	0.054 (0.003–0.993)	0.049	0.189 (0.008–4.549)	0.305	0.205 (0.009–4.520)	0.315
MVC	2.368 (1.050–5.341)	0.038	1.310 (0.562–3.051)	0.532	1.398 (0.600–3.261)	0.437
CRP	1.133 (1.063–1.208)	<0.001	1.087 (1.016–1.162)	0.015	1.096 (1.026–1.172)	0.007
Uric acid	1.393 (1.216–1.597)	<0.001	1.337 (1.142–1.565)	<0.001	-	-
Albumin	0.377 (0.254–0.558)	<0.001	0.396 (0.251–0.623)	<0.001	-	-
UAR	2.724 (1.999–3.713)	<0.001	-	-	2.478 (1.779–3.453)	<0.001

* The variables with a *p*-value of less than 0.05 in the univariate analysis were incorporated into the multivariate Cox regression analysis by using the Enter method. Abbreviations: VDH, vascular disease history; AVA, aortic valve area; MVC, Major vascular complications; UAR, uric acid to albumin ratio.

## Data Availability

Data are available upon reasonable request.

## References

[1] Supino P.G., Borer J.S., Preibisz J., Bornstein A. (2006). The Epidemiology of Valvular Heart Disease: A Growing Public Health Problem. Heart Fail. Clin..

[2] Otto C.M., Kuusisto J., Reichenbach D.D., Gown A.M., O’Brien K.D. (1994). Characterization of the early lesion of ‘degenerative’ valvular aortic stenosis. Histological and immunohistochemical studies. Circulation.

[3] Aronow W.S., Ahn C., Kronzon I., Goldman M.E. (2001). Association of coronary risk factors and use of statins with progression of mild valvular aortic stenosis in older persons. Am. J. Cardiol..

[4] Masson J.-B., Kovac J., Schuler G., Ye J., Cheung A., Kapadia S., Tuzcu M.E., Kodali S., Leon M.B., Webb J.G. (2009). Transcatheter Aortic Valve Implantation: Review of the Nature, Management, and Avoidance of Procedural Complications. JACC Cardiovasc. Interv..

[5] Hioki H., Watanabe Y., Kozuma K., Yamamoto M., Naganuma T., Araki M., Tada N., Shirai S., Yamanaka F., Higashimori A. (2018). Effect of Serum C-Reactive Protein Level on Admission to Predict Mortality After Transcatheter Aortic Valve Implantation. Am. J. Cardiol..

[6] Ames B.N., Cathcart R., Schwiers E., Hochstein P. (1981). Uric acid provides an antioxidant defense in humans against oxidant- and radical-caused aging and cancer: A hypothesis. Proc. Natl. Acad. Sci. USA.

[7] Ekaidem I.S., Usoh I.F., Akpanabiat M.I., Uboh F., Akpan H.D. (2014). Urate Synthesis and Oxidative Stress in Phenytoin Hepatotoxicity: The Role of Antioxidant Vitamins. Pak. J. Biol. Sci..

[8] Ya B.-L., Liu Q., Li H.-F., Cheng H.-J., Yu T., Chen L., Wang Y., Yuan L.-L., Li W.-J., Liu W.-Y. (2018). Uric Acid Protects against Focal Cerebral Ischemia/Reperfusion-Induced Oxidative Stress via Activating Nrf2 and Regulating Neurotrophic Factor Expression. Oxidative Med. Cell. Longev..

[9] Peng F., Yang Y., Liu J., Jiang Y., Zhu C., Deng X., Hu X., Chen X., Zhong X. (2012). Low antioxidant status of serum uric acid, bilirubin and albumin in patients with neuromyelitis optica. Eur. J. Neurol..

[10] Yang D., Su Z., Wu S., Bi Y., Li X., Li J., Lou K., Zhang H., Zhang X. (2016). Low antioxidant status of serum bilirubin, uric acid, albumin and creatinine in patients with myasthenia gravis. Int. J. Neurosci..

[11] Peters T. (1985). Serum Albumin. Adv. Protein Chem..

[12] Kappetein A.P., Head S.J., Généreux P., Piazza N., Van Mieghem N.M., Blackstone E.H., Brott T.G., Cohen D.J., Cutlip D.E., Van Es G.-A. (2012). Updated standardized endpoint definitions for transcatheter aortic valve implantation: The Valve Academic Research Consortium-2 consensus document. Eur. Heart J..

[13] Rutkovskiy A., Malashicheva A., Sullivan G., Bogdanova M., Kostareva A., Stensløkken K., Fiane A., Vaage J. (2017). Valve Interstitial Cells: The Key to Understanding the Pathophysiology of Heart Valve Calcification. J. Am. Heart Assoc..

[14] Katkat F., Kalyoncuoglu M., Ozcan S., Tugrul S., Abanus H., Ince O., Balli M., Sahin I., Okuyan E. (2022). C-Reactive Protein to Albumin Ratio as A Novel Inflammatory-Based Marker for 30-Day Mortality in Patients Undergoing Transcatheter Aortic Valve Replacement. Rev. Bras. Cir. Cardiovasc..

[15] Cybularz M., Wydra S., Berndt K., Poitz D.M., Barthel P., Alkouri A., Heidrich F.M., Ibrahim K., Jellinghaus S., Speiser U. (2021). Frailty is associated with chronic inflammation and pro-inflammatory monocyte subpopulations. Exp. Gerontol..

[16] Hu B., Yang X.-R., Xu Y., Sun Y.-F., Sun C., Guo W., Zhang X., Wang W.-M., Qiu S.-J., Zhou J. (2014). Systemic Immune-Inflammation Index Predicts Prognosis of Patients after Curative Resection for Hepatocellular Carcinoma. Clin. Cancer Res..

[17] Xiong Z., Zhu C., Qian X., Zhu J., Wu Z., Chen L. (2011). Predictors of clinical SYNTAX score in coronary artery disease: Serum uric acid, smoking, and Framingham risk stratification. J. Invasive Cardiol..

[18] Lin G.-M., Li Y.-H., Zheng N.-C., Lai C.-P., Lin C.-L., Wang J.-H., Jaiteh L.E., Han C.-L. (2013). Serum uric acid as an independent predictor of mortality in high-risk patients with obstructive coronary artery disease: A prospective observational cohort study from the ET-CHD registry, 1997–2003. J. Cardiol..

[19] Stinefelt B., Leonard S.S., Blemings K.P., Shi X., Klandorf H. (2005). Free radical scavenging, DNA protection, and inhibition of lipid peroxidation mediated by uric acid. Ann. Clin. Lab. Sci..

[20] Yu M.-A., Sánchez-Lozada L.G., Johnson R.J., Kang D.-H. (2010). Oxidative stress with an activation of the renin–angiotensin system in human vascular endothelial cells as a novel mechanism of uric acid-induced endothelial dysfunction. J. Hypertens..

[21] Goh S.L., De Silva R.P., Dhital K., Gett R.M. (2014). Is low serum albumin associated with postoperative complications in patients undergoing oesophagectomy for oesophageal malignancies?. Interact. Cardiovasc. Thorac. Surg..

[22] Kim H.J., Lee H.W. (2013). Important predictor of mortality in patients with end-stage liver disease. Clin. Mol. Hepatol..

[23] Gupta D., Lis C.G. (2010). Pretreatment serum albumin as a predictor of cancer survival: A systematic review of the epidemiological literature. Nutr. J..

[24] Wada H., Dohi T., Miyauchi K., Doi S., Naito R., Konishi H., Tsuboi S., Ogita M., Kasai T., Okazaki S. (2017). Independent and combined effects of serum albumin and C-reactive protein on long-term outcomes of patients undergoing percutaneous coronary intervention. Circ. J..

[25] Fairclough E., Cairns E., Hamilton J., Kelly C. (2009). Evaluation of a modified early warning system for acute medical admissions and comparison with C-reactive protein/albumin ratio as a predictor of patient outcome. Clin. Med..

[26] Okuno T., Koseki K., Nakanishi T., Sato K., Ninomiya K., Tomii D., Tanaka T., Sato Y., Horiuchi Y., Koike H. (2019). Evaluation of objective nutritional indexes as predictors of one-year outcomes after transcatheter aortic valve implantation. J. Cardiol..

[27] Özgür Y., Akın S., Yılmaz N.G., Gücün M., Keskin Ö. (2021). Uric acid albumin ratio as a predictive marker of short-term mortality in patients with acute kidney injury. Clin. Exp. Emerg. Med..

[28] de Melo P.H.M.C., Modolo R. (2021). The Role of Inflammation in Post-TAVI Outcomes. Arq. Bras. Cardiol..

[29] Çakmak E., Bayam E., Çelik M., Kahyaoğlu M., Eren K., Imanov E., Karagöz A., İzgi İ.A. (2020). Uric Acid-to-Albumin Ratio: A Novel Marker for the Extent of Coronary Artery Disease in Patients with Non-ST-Elevated Myocardial Infarction. Pulse.

[30] Li S., Chen H., Zhou L., Cui H., Liang S., Li H. (2022). The uric acid to albumin ratio: A novel predictor of long-term cardiac mortality in patients with unstable angina pectoris after percutaneous coronary intervention. Scand. J. Clin. Lab. Investig..

